# Effects of lower limb resistance exercise on muscle strength, physical fitness, and metabolism in pre-frail elderly patients: a randomized controlled trial

**DOI:** 10.1186/s12877-021-02386-5

**Published:** 2021-07-30

**Authors:** Xiaoxing Lai, Lin Bo, Hongwei Zhu, Baoyu Chen, Zhao Wu, Hongdi Du, Xiaopeng Huo

**Affiliations:** 1 Department of Health Care, Peking Union Medical College Hospital, Beijing, 100730 China; 2 Nursing Department, Peking Union Medical College Hospital, Beijing, 100730 China

**Keywords:** Elderly, Frailty, Exercise, Muscle strength, Physical fitness, Metabolism

## Abstract

**Background:**

Few studies examined interventions in frail elderly in China, while the awareness of applying interventions to prevent frailty in pre-frail elderly is still lacking. This study aimed to explore the effects of lower limb resistance exercise in pre-frail elderly in China.

**Methods:**

This was a randomized controlled trial of patients with pre-frailty. The control group received routine care, while the exercise group received a 12-week lower limb resistance exercise based on routine care. The muscle strength in the lower limbs, physical fitness, and energy metabolism of the patients was evaluated at admission and after 12 weeks of intervention.

**Results:**

A total of 60 pre-frail elderly were included in this study. The patients were divided into the exercise group (*n* = 30) and control group (*n* = 30) by random grouping. There were 17 men and 13 women aged 65.3 ± 13.4 in the exercise group, and 15 men and 15 women aged 67.6 ± 11.9 years in the control groups. The Barthel index was 80.3 ± 10.6 and 85.1 ± 11.6, respectively. The characteristics of the two groups were not significantly different before intervention (all *p* > 0.05). The results of repeated measurement ANOVA showed that there was statistically significant in crossover effect of group * time (all *p* < 0.05), that is, the differences of quadriceps femoris muscle strength, 6-min walking test, 30-s sit-to-stand test, 8-ft “up & go” test, daily activity energy expenditure and metabolic equivalent between the intervention group and the control group changed with time, and the variation ranges were different. The main effects of time were statistically significant (all *p* < 0.05), namely, femoris muscle strength, 6-min walking test, 30-s sit-to-stand test, 8-ft “up & go” test, daily activity energy expenditure and metabolic equivalent of the intervention group and the control group were significantly different before and after intervention. The main effects of groups were statistically significant (*p* < 0.05), namely, femoris muscle strength, 6-min walking test, 30-s sit-to-stand test, daily activity energy expenditure and metabolic equivalent before and after intervention were significantly different between the intervention group and the control group, while there was no significant differences in 8-ft “up & go” test between groups.

**Conclusion:**

Lower limb resistance exercise used for the frailty intervention could improve muscle strength, physical fitness, and metabolism in pre-frail elderly.

**Trial registration:**

ChiCTR, ChiCTR2000031099. Registered 22 March 2020, http://www.chictr.org.cn/edit.aspx?pid=51221&htm=4

## Background

Frailty is a clinical syndrome of increased vulnerability and functional impairment due to cumulative decline among multiple body systems, which increases the risk of a variety of adverse health outcomes, including falls, hospitalization, and death [1–3]. While frailty does not refer specifically to chronological age or disability, advanced age increases the risk of developing frailty [2, 3]. The prevalence of frailty has been reported to be 15% in community-dwelling adults > 65 years old in the United States of America [4], and the worldwide prevalence varies between 3.5 and 27.3% [3]. The risk factors for frailty and/or functional decline include advanced age, polypharmacy, lack of regular exercise, poverty and/or isolation, poor nutrition, malnutrition, and/or weight loss, cognitive impairment, and medical and/or psychiatric comorbidities [2, 3].

Fried et al. defined the phenotypes of frailty using five criteria [5]: 1) generalized weakness; 2) loss of endurance; 3) decreased mobility; 4) spontaneous body mass loss; 5) decreased activity. Patients meeting one or two of the items are considered as pre-frail, while patients meeting ≥3 items are considered as frail [5]. Pre-frailty is considered as a state in which the health condition is relatively poor but has not yet reached the state of overt frailty [4, 5].

Due to its reversibility, pre-frailty is considered a key condition that must be recognized and addressed in clinical practice [6]. Exercise is an effective method for delaying or even reversing the progression of frailty and improving frailty components [6–14]. One of the important indicators of frailty is the decline in muscle strength and endurance [9]. The decline in muscle strength occurs due to the reduction of muscle quality. Concurrently, the elderly experience dysfunction and multiple diseases as part of the aging process, which is often accompanied by inflammation. Inflammatory cytokines promote skeletal muscle catabolism, inhibit muscle protein synthesis, reduce skeletal muscle mass and muscle strength, and then lead to decreased activity or even disability [10]. An umbrella review of seven systematic reviews examined exercise interventions in pre-frailty and frailty [15]. Resistance exercise can significantly increase the levels of insulin-like growth factors-1 (IGF-1) and IGF-1 receptor (IGF-1R) and can lead to better effects in preventing age-related muscle atrophy compared to aerobic exercises [16]. Unfortunately, the number and compliance of the elderly receiving muscle strength exercise are still low [17, 18]. Progressive resistance strength training for 4 months could increase the muscle mass by 2–9% in frail elderly and increase their walking speed [19].

Physical fitness refers to the human body’s ability to safely and independently carry out daily activities without feeling tired, which is closely related to reducing fall risk and enhancing cognitive ability. In 2000, the State Sports General Administration of China published the “National Fitness Measurement Standard Manual”, with a special section dedicated to the elderly. The main shortcomings of Chinese physical fitness tests for the elderly are few test items, short age range, and simple evaluation methods. Thus, a comprehensive, scientific, and safe test with good reliability and validity for the physical fitness of the Chinese elderly is needed.

Walking is the basic guarantee that the elderly can live independently and one of the important indicators to evaluate their frailty. There were few Intervention studies on the pre-frail and frail elderly, especially on the walking ability and physical fitness of the elderly. Therefore, this study aimed to investigate the effects of lower limb resistance exercise on muscle strength, physical fitness, and energy metabolism in pre-frail elderly, as this could be a relatively simple method for postponing the development of frailty and improving the quality of life in the elderly.

## Methods

### Study design and sampling

This was a randomized controlled trial of patients with pre-frailty. Convenience sampling was performed to enroll elderly hospitalized at the Health care Department of Peking Union Medical College Hospital between April 2020 and September 2020.

The study was approved by the Ethics Committee of Peking Union Medical College Hospital (No.JS-2264). All participants signed the informed consent form after the investigator explained the study.

### Inclusion and exclusion criteria of participants and grouping

The inclusion criteria were following: 1) volunteered to participate in this study, 2) ≥60 years of age, 3) met the diagnostic criteria of pre-frailty described by Fried et al. [5], 4) Karnofsky Performance Status (KPS) score > 80 points, and 5) could take care for themselves. The exclusion criteria were 1) with cognitive disorders (Montreal Cognitive Assessment (MoCA) score < 26 points) and could not cooperate with the study, 2) medical records reporting neuromuscular disorders, skeletal and joint abnormality, or severe cardiac insufficiency; 3) were already doing regular physical activities (less than three times per week, and 30 min every time); 4) underwent surgery within 1 month; 5) unsuitable to participate in the research according to researchers.

The sample size was calculated (http://powerandsamplesize.com/Calculators/) as 31 per group (considering α of 0.05, power of 80%, and intergroup difference of 1.3 kg in the strength of the quadriceps femoris muscle), including a loss to follow-up rate of 20%. Group division: to divide qualified subjects of 60 patients into exercise group and control group with 31 patients in the ratio of 1: 1 by means of random number table. A researcher who was blinded of assignment to interventions enrolled participants, and another researcher assigned participants to interventions. The subjects were blinded of assignment to interventions.

### Routine treatment and care

Routine treatment and care for patients’ diseases (such as oral medication, intravenous medication, aerosol inhalation therapy and so on) were applied for the patients in both groups according to their specific conditions. Patients in both groups received exercise guidance, such as face-to-face exercise education that explained the necessity and effective methods of exercise for elderly patients. The participants were followed by telephone calls once every month, and their health conditions, living conditions, and recent exercises (including intensity, frequency, and duration) were enquired during follow-up.

### Lower limb resistance exercise

For the participants in the exercise group, 12 weeks’ lower limb resistance exercise was applied as part of the exercise intervention, in addition to the routine treatment and care. The intervention was started within the week after hospital admission and lasted for 12 weeks. If the participants were discharged during the intervention, the intervention was continued by follow-up. The specific lower limb resistance exercise strategy for the elderly was specially designed for this study by the rehabilitation physicians in our research team according to the physiological characteristics of the pre-frail elderly and consisted of three steps of standardizing exercise process (warm-up, training, and relaxation). An active low-resistance exercise consisting of five sections was applied in this study. Such an exercise was enough to sufficiently exercise the lower limb muscle group, including the quadriceps femoris, gluteus maximus, and gastrocnemius muscles, and to allow for the wide range of movement of hip joints knee joints, and ankles. According to the participants’ conditions, after the elderly people got used to the exercise, wrist-mounted sandbags of 0.5–1 kg were attached to their ankles during each training. The intervention in the first week was performed in the rehabilitation center of the Rehabilitation Department. In contrast, the consequent interventions from the second week to the end were performed in the ward.

Side lift of the leg was performed to enhance the hips, buttocks, and thighs and improve balance. The participants were asked to stand behind a secure chair, hold the chair back to maintain balance, exhale slowly, lift one leg to one side, and keep the back straight, with the foot tip downward, while the standing leg was appropriately bent. The participants were asked to maintain the posture for 1 s, then slowly inhale and slowly put down the leg. Each leg was exercised 10–15 times.

Back lift of the leg was performed to enhance the strength of the buttocks and back and improve balance. The participants were asked to stand behind a secure chair, hold the chair back to maintain balance, exhale slowly, and lift one leg slowly backward, without bending the knee and toes if possible. The participants tried not to lean forward while the standing leg was slightly bent. The participants were asked to maintain this posture for 1 s, then to slowly inhale and slowly put down the leg. Each leg was exercised 10–15 times.

For knee bending, the participants were asked to stand behind a secure chair, hold the chair back to maintain balance, straighten one leg backward and lift it without bending the knees and toes if possible. The participants were asked to slowly inhale and exhale, bend the knee to move the foot as close to the buttocks as possible while not moving the buttocks. The standing leg was slightly bent. The participants were asked to maintain such posture for 1 s, then to slowly inhale and put down the leg. Each leg was exercised 10–15 times.

Knee extension was performed to enhance the strength of the legs and protect the knee joint. The participants were asked to sit in a secure chair, with the backrest on the chair back. The forefeet and toes were relaxed and put on the floor. A towel roll was placed at the chair’s edge and under the thigh to support the thigh. The participants were asked to breathe deeply, then to slowly extend one leg forward and try to straighten the leg as much as possible and prevent the knee from bending. The foot was bent to make the toes upward. The participants were asked to maintain such posture for 1 s, then to slowly inhale and slowly put down the leg. Each leg was exercised 10–15 times.

Standing on tiptoes was performed to enhance the strength of calves and ankles and make walking more stable. The participants were asked to stand behind a secure chair, hold the chair back to maintain balance, and spread their feet to the shoulders’ width. They were then asked to inhale and slowly exhale and to slowly stand on tiptoes as high as possible. The posture was maintained for 1 s, after which the participants were asked to slowly inhale and slowly put down the leg. Each leg was exercised for 10–15 times. The participants were trained three times every week (on Mondays, Wednesdays, and Fridays); there were three groups in each training, and each group included the whole five movements. Each training lasted for 30 min. The intervention lasted 12 weeks. The number of sets and repetitions in training volume and intensity were identical. The nurses were asked to demonstrate the exercises, explain the significant points of the movements, help correct mistakes, and ensure the participants’ safety. The Borg Rating of Perceived Exertion Scale [20] was used to evaluate exercise intensity. The participants were asked to maintain the self-perceived exertion degree at 11–13 points, which meant that the exercise was a little intense.

The exercise was suspended for participants with the following conditions: unstable blood pressure or blood glucose, dizziness, shortness of breath, pain or constriction in the chest, palpitation, thrombosis, muscle pain, unhealed wound, joint swelling, and retinal hemorrhage or detachment. The exercise was immediately stopped if the patients reported pain or constriction in the chest/neck/shoulder or arms, dizziness or nausea, or muscle spasm during the exercise.

### Home exercises for discharged participants

One-on-one education held by nurses was provided to each participant before discharge, and the participants were required to consent that they understood. At the same time, the participants’ families or companions also participated in the education to ensure that the participants could continue the exercise correctly after discharge. Reminders and schedules were sent to the participants by nurses when necessary. Besides, the nurses also provide guidance and follow-up to the participants by WeChat, telephone call, or home visit on Mondays, and then the follow-up data were recorded. The participants were required to upload videos of their exercises on Fridays and complete the exercise interventions after discharge. Efforts were made to ensure that the participants could continue the correct and standard exercises after discharge. The participants returned to the hospital after a total of 12 weeks of intervention for evaluation.

### Measurement of outcomes

A Convenient Digital Muscle Strength Measuring Instrument (Series EKK; MARK-10, Copiague, NY, USA), which could quantitatively measure and then display the muscle strength as digits (kg), was used to measure the muscle strength in both lower limbs. The Quadriceps femoris muscle is the lower limb’s major muscle and the largest and most powerful muscle of the body, and also an important muscle for maintaining knee joint stability, walking, and running. Therefore, the strength of the quadriceps femoris muscle was measured in this study. The patients were placed in the sitting position, with both feet naturally resting on the floor and knee joints bent at a 90° angle. The muscle strength measuring device was uniformly placed at the lower edge of the knee. The participants were asked to extend the knee, and the muscle strength of the quadriceps femoris muscle was displayed as digits. The measurement was repeated three times, and the average value was calculated. All researchers who participated in the measurement have been trained on the application method of the instrument by the engineer of muscle strength measuring instrument manufacturer, and two researchers formed a group when measuring to make sure all of the measurements were correctly conducted. The subjects were familiarized with the purpose, operation process, and precautions of the test, and the researchers and the subjects worked together to practice the test before the measurement.

Physical fitness was measured by the Senior Fitness Test (SFT) developed by Rikli et al. [21, 22]. The SFT can be used to evaluate various fitness levels in healthy or frail elderly peoples aged 60–90 years and living independently. Among six parts the test includes three that are specific for lower limb exercises.

The 6-min walking test (6MWT) was performed along a 30-m corridor. The patient received an explanation of what was required in the test and was told to walk as far as possible in 6 min without running. After the start signal, the tested participant walked as fast as possible, and the distance walked within 6 min was recorded (under the conditions of being physically comfortable). For the 30-s sit-to-stand test (m30STS), the participants sat in a chair, with both feet resting on the floor. After hearing the start signal, the patient stood up and then returned to the sitting position. The total number of sit-to-stand completed within 30 s was recorded. For the 8-ft “up & go” test, the patients were sitting in a chair. After hearing the start signal, the patients stood up, walked as fast as possible to the marker 8 ft away, and then walked back to sit in the chair. The time (seconds) that the patients used in this process were recorded. Before the formal tests, the researcher showed the participants how to do the test correctly, after which the participants did a pre-test to familiarize themselves with the tests.

A wireless wGT3X-BT 3-axis ActiGraph (wGT3X-BT, ActiGraph, LLC, Pensacola, FL, USA) was used to measure energy metabolism. The ActiGraph is a friendly, watch-sized wearable device that is convenient to wear and has data storage and recording capability. The device was connected to the ActiLife software platform (Version 6.13.4, ActiGraph, LLC, Pensacola, FL, USA), and the data could be output and analyzed. In this study, the activity expenditure and metabolic equivalent (MET) rate were measured by the device. The patients were asked to wear the device for 48 h twice (before and after intervention), and the average value of the two 24-h values was calculated.

### Follow-up

Follow-up included patients with less than 12 weeks of hospitalization to ensure the completion of the 12 weeks exercise intervention. Archives of exercise management were established to collect exercise records after discharge. Participant patients were ruled out from the study if they did not upload videos of exercises for 2 weeks or did not participate for 2 weeks and if they or their families confirmed not participating in the exercise intervention.

### Quality control

The nurses who participated in this study were uniformly trained by the investigators, ensuring consistency and standardization of the exercise intervention.

### Data collection

The investigators in charge of data collection were blinded to grouping throughout the study. The participants’ general characteristics included sex, age, educational level, types of chronic diseases, and activities of daily living (ADL). The ADL was evaluated by the Barthel activities of daily living index, which classifies the ADL as independent (100 points), slightly dependent (75–95 points), moderately dependent (50–70 points), highly dependent (25–45 points), and entirely dependent (0–20 points) [23]. The muscle strength in the lower limbs, physical fitness, and energy metabolism of the patients was evaluated at admission and after 12 weeks of intervention.

### Statistical analysis

SPSS 25.0 (IBM, Armonk, NY, USA) was used for statistical analysis. The quantitative data were firstly tested for normal distribution, after which the homogeneity of variance was tested. If not, the homogeneity was achieved by variable transformation. For quantitative data, means and standard deviations ($\overline{x}$ *±s*) were used for description; the independent *t*-test was used for comparisons between the two groups. For enumeration data, frequencies and percentages (n, %) were used for description, and the chi-square test was used for comparisons between the two groups. The repeated measurement data were compared between each time points by repeated measurement ANOVA (RM—ANOVA). All statistical analyses were two-sided. A *p*-values < 0.05 were considered statistically significant.

## Results

### Characteristics of the participants

During the study, one participant in the exercise group was excluded for not cooperating with the study. One participant in the control group dropped off due to myocardial infarction. The participants were 65.3 ± 13.4 and 67.6 ± 11.9 years of age, while the age of the pre-frail elderly in this study ranged from 60 to 79 years old. There were 56.7 and 50.0% males in the exercise and control groups, respectively. The educational level of most subjects was a primary school and middle school. In addition, the Barthel index was 80.3 ± 10.6 and 85.1 ± 11.6 in the exercise and control groups, respectively. The most common diseases among pre-frail elderly in this study were: cardiovascular and cerebrovascular disease, respiratory system disease, and diabetes. The number of taken drugs were 3.5 ± 0.5 and 3.2 ± 0.7 pills in the exercise and control groups, respectively. The general characteristics did not significantly differ between the two groups (all *p* > 0.05)(Table 1).

**Table 1:** Characteristics of the participants

Indicator	Exercise group (*n* = 30)	Control group (*n* = 30)	*p*
Sex, n (%)			0.605
Male	17 (56.7)	15 (50.0)	
Female	13 (43.3)	15 (50.0)	
Age (years)	65.3 ± 13.4	67.6 ± 11.9	0.482
Educational level, n (%)			0.447
Illiteracy	2 (6.7)	3 (10.0)	
Primary school	11 (36.7)	13 (43.3)	
Middle school	12 (40.0)	10 (33.3)	
Junior college or higher	5 (16.6)	4 (13.4)	
Barthel activities of daily living (points)	80.3 ± 10.6	85.1 ± 11.6	0.094
n (%)			
100 points: independent	2 (6.7)	3 (10.0)	1.000
75–95 points: slightly dependent	28 (93.3)	27 (90.0)	
50–70 points: moderately dependent	0	0	
25–45 points: highly dependent	0	0	
0–20 points: completely dependent	0	0	
Comorbidities, n (%)			0.923
Cardiovascular and cerebrovascular disease	9 (30.0)	8 (26.6)	
Respiratory system disease	6 (20.0)	5 (16.7)	
Diabetes	5 (16.7)	4 (13.3)	
Digestive system disease	4 (13.3)	3 (10.0)	
Nervous system disease	3 (10.0)	5 (16.7)	
Others	3 (10.0)	5 (16.7)	
No. of the drugs(pills/capsules)	3.5 ± 0.5	3.2 ± 0.7	0.061
Hospital stay (weeks)	6.9 ± 0.7	7.1 ± 0.7	0.254
Duration of intervention after discharge (weeks)	4.1 ± 0.9	4.6 ± 0.8	0.033
Ratio of hospital stay/outside hospital stay	2.2 ± 0.7	2.3 ± 0.8	0.554

### Physical indicators of exercise

The results of RM—ANOVA showed that there was statistically significant in crossover effect of group * time (all *p* < 0.05) (Table 2), that is, the differences of quadriceps femoris muscle strength, 6-min walking test, 30-s sit-to-stand test, 8-ft “up & go” test, daily activity energy expenditure and metabolic equivalent between the intervention group and the control group changed with time, and the variation ranges were different. The main effects of time were statistically significant (all *p* < 0.05) (Table 2), namely, femoris muscle strength, 6-min walking test, 30-s sit-to-stand test, 8-ft “up & go” test, daily activity energy expenditure and metabolic equivalent of the intervention group and the control group were significantly different before and after intervention. The main effects of groups were statistically significant (*p* < 0.05) (Table 2), namely, femoris muscle strength, 6-min walking test, 30-s sit-to-stand test, daily activity energy expenditure and metabolic equivalent before and after intervention were significantly different between the intervention group and the control group, while there was no significant difference in 8-ft “up & go” test between the intervention group and the control group.

**Table 2:** Comparison of indicators between the two groups before and after the intervention

Group	Exercise group, *n* = 30		Control group, *n* = 30		Groups*Time		Time		Groups	
					*F*	*P*	*F*_*1*_	*P*_*1*_	*F*_*2*_	*P*_*2*_
Time	Before	After	Before	After						
Quadriceps femoris muscle strength (kg)	5.76 ± 0.8	8.04 ± 0.96	6.11 ± 1.15	6.04 ± 0.92	58.172	<0.001	51.373	<0.001	18.112	<0.001
6-min walking test (m)	548.04 ± 74	653.18 ± 56.79	559.68 ± 67.05	548.8 ± 63.87	22.895	<0.001	15.113	<0.001	15.260	<0.001
30-s sit-to-stand test (times)	12.87 ± 3.31	17.21 ± 3.95	13.16 ± 4.1	13.43 ± 4.22	10.961	<0.001	14.035	<0.001	4.741	0.034
8-ft “up & go” test (s)	5.80 ± 0.83	4.71 ± 0.8	5.51 ± 0.49	5.29 ± 0.67	23.777	<0.001	53.186	<0.001	0.804	0.374
Daily activity energy expenditure (kcal)	225.85 ± 38.2	405.66 ± 47.01	218.21 ± 35.96	230.03 ± 40.17	132.717	<0.001	172.723	<0.001	148.759	<0.001
Metabolic equivalent	2.33 ± 0.29	4.51 ± 0.45	2.43 ± 0.36	2.56 ± 0.5	239.985	<0.001	305.844	<0.001	125.497	<0.001

## Discussion

Few studies examined interventions in frail elderly in China, while the awareness of applying interventions to prevent frailty in pre-frail elderly is still lacking. Therefore, the aim of this study was to explore the effects of lower limb resistance exercise in the pre-frail elderly. Our results strongly suggest that lower limb resistance exercise for the frailty intervention could improve muscle strength, physical fitness, and the pre-frail elderly’s metabolism.

### Lower limb resistance exercise could enhance the muscle strength of the pre-frail elderly patients

Our results showed that compared with the control group, the strength in the quadriceps femoris muscle significantly increased in the exercise group after intervention. An active low-resistance exercise was applied in this study, and all participants completed five sessions of exercise, which was apparently enough to sufficiently exercise the lower limb muscle group, including the quadriceps femoris, gluteus maximus, gastrocnemius muscles, and to allow the wide range of movement of hip joints, knee joints, and ankles. To suit the physical fitness of pre-frail elderly participants, the exercise intensity was gradually increased according to the exercise tolerance. Sandbags of 0.5–1 kg were attached to the participants’ ankles during the exercise to increase the intensity of resistance exercise further and increase the muscle strength. Previous studies have shown that resistance exercise can induce muscle movements, increase muscle strength, and maintain soft tissues’ length and flexibility [6–14]. Progressive resistance exercise for 4 months can increase the muscle mass by 2–9% and significantly improve the walking speed in frail elderly [19]. The resistance exercises include accelerating blood circulation and metabolism of skeletal muscles and increase skeletal muscle strength [19]. Such processes mainly rely on remodeling (changes of muscle mass and volume) and changing the molecular components (such as the dominant changes of the expression of myoglobulin subtypes) of muscle fibers, consequently improving the growth of skeletal muscles and inducing a rapid increase in strength [24, 25].

### Lower limb resistance exercise could improve the physical fitness in pre-frail elderly patients

Our results demonstrated that after 12 weeks of intervention, the SFT score was significantly higher in the exercise group than in the control group, thus suggesting that lower limb resistance exercise could effectively improve the capability of performing physical activities and even reverse the state of pre-frailty in elderly. Physical fitness refers to subjects’ capability to safely and independently perform daily activities without feeling physical fatigue [21, 22]. The lower limb resistance exercise designed in this study could relatively comprehensively enhance the strength of the hip, buttocks, thighs, calves, and ankles, which in turn made walking more stable and effortless and improved the activity capability in the elderly. For instance, the leg’s side-lift in the first session enhanced the strength of the hip, buttocks, and thighs and protected the femur and femoral neck. Such exercises do not only improve balance capability but also effectively prevent fractures caused by falling backward. Faber et al. [26] found that the interventional effects were higher in pre-frail patients than in frail patients; besides, pre-frail patients had slighter fatigue and could better cooperate with the intervention. Previous studies have demonstrated that physical fitness can be used as a useful early indicator for assessing ADL in elderly individuals [6–14], and the capability of physical activities was positively associated with the quality of lives, which was in agreement with the findings of this study.

### Lower limb resistance exercise could increase the energy metabolism of the pre-frail elderly patients

Our results showed that after 12 weeks of intervention, the energy expenditure for daily activities in the exercise group was significantly higher than before the intervention and higher than in the control group after intervention, thus suggesting that lower limb resistance exercise could improve the metabolism of pre-frail elderly. When analyzed from the physiological and biochemical aspects, it can be speculated that resistance exercise induces corresponding adaptive morphological and functional changes in the skeletal muscles, thus improving the muscular metabolism, regulating lipid metabolism, and increasing the metabolism capability. Increasing physical activity levels is the preferred method for balancing energy metabolism [8]. Muscle tissues, which are important organs for activities, also have an essential role in the body’s energy metabolism. Regular resistance exercise could increase the strength and size of muscles and lean body weight, improve basal metabolic rate (BMR), promote fat metabolism in muscles, and increase insulin activity in muscles [27]. Intramyocellular triglycerides (IMTG) exist in muscle fibers as lipid droplets and can rapidly be hydrolyzed and oxidized and provide energy for muscular activities [28]. Resistance exercise is one of the best exercises for promoting IMTG metabolism. In the study performed by Harber et al. [28], six overweight women with high body fat percentage received six sessions of knee joint resistance exercises at the level of 70% 1RM, after which a biopsy of the vastus lateralis muscle was obtained at 1 min and 2 h after exercise. Their findings showed that the IMTG was reduced by 40% at 1 min and 2 h after the resistance exercise. In this study, lower limb resistance exercise was used, and the BMR was increased, possibly by enhancing oxidation, catecholamine level, and protein synthesis, and consequently increasing the MET.

### Wearable technology could be used for the health management of the elderly

Novel wearable technology (WT) is a potent tool for individualized medicine that can break down the temporal and spatial barriers and more safely and reliably collect data from subjects and provide a model to analyze the data more precisely [29, 30]. In this study, we used the wireless 3-axis ActiGraph, which measures the physiological activities of elderlies with a motion sensor at the maximum sampling frequency of 100 Hz, thus collecting high-quality health data including movement counts, energy expenditure, MET, vector magnitude, and step count that help to analyze the exercise effects in the participants [31]. Wearable medical devices are currently widely used to adjust the freezing gait of Parkinson’s disease patients, monitor blood glucose, assist patients with limb activity disorder, and orient patients with Alzheimer’s disease [32]. Nevertheless, few studies investigated the application of such devices in monitoring the exercises of the elderly. The awareness rate of wearable devices in China is as high as 54.6%. Still, the actual utilization rate is only 2.9% [33], which could be because most people are still not aware of the effect wearable devices have on health promotion. Therefore, such wearable devices have bright prospects and advantages in managing health exercises in the elderly.

### Limitations

Enrollment was based on convenience sampling, and no randomization was performed. Of course, due to the nature of the intervention, double-blinding was not possible, and only the data assessors were blind. The follow-up time in this study was relatively short, and no data about prognosis were collected. Energy expenditure was analyzed, but no biochemical markers were assessed to determine the exact mechanisms. Further studies are still needed to validate the relationship between resistance exercise and disability.

## Conclusion

Lower limb resistance exercise might be a safe and effective method for rehabilitation exercise in the pre-frail elderly. When used within an appropriate range, it can increase the strength of lower limb muscle. SFT is an effective and simple method to measure the fitness status of the elderly in China. Wearable devices were suitable for monitoring the health status of the elderly. Frailty is a dynamic process, and early recognition and frailty intervention are essential for delaying or even reversing frailty. Therefore, regular resistance exercise can delay aging and enhance physical condition in pre-frail elderly.

## Data Availability

The datasets generated and/or analyzed during the current study are not publicly available due to the need to protect patient privacy but are available from the corresponding author on reasonable request.

## Abbreviations

IGF-1: Insulin-like growth factors-1
IGF-1R: Insulin-like growth factors-1receptor
SFT: Senior fitness test
KPS: Karnofsky Performance Status
MoCA: Montreal Cognitive Assessment
6MWT: 6-min walking test
m30STS: 30-s sit-to-stand test
IPAQ-SF: International Physical Activity Questionnaire-Short Form
ADL: Activities of daily living
BMR: Basal metabolic rate
IMTG: Intramyocellular triglycerides
WT: Wearable technology
